# O026. An abnormal transduction of the chromatic stimuli from the outer to the inner retinal layers may contribute to cause photophobia in migraine

**DOI:** 10.1186/1129-2377-16-S1-A54

**Published:** 2015-09-28

**Authors:** Gianluca Coppola, Luisella Corso, Antonio Di Renzo, Antonello Fadda, Francesco Martelli, Cherubino Di Lorenzo, Vincenzo Parisi, Jean Schoenen, Benedetto Falsini, Francesco Pierelli

**Affiliations:** 1 Department of Neurophysiology of Vision and Neuro-ophthalmology, G.B. Bietti Foundation IRCCS, Rome, Italy; 2 Department of medico-surgical sciences and biotechnologies, “Sapienza” University of Rome Polo Pontino, Latina, Italy; 3 Dipartimento Tecnologie e Salute, Istituto Superiore di Sanità, Rome, Italy; 4 Don Carlo Gnocchi Onlus Foundation, Milan, Italy; 5 Headache Research Unit, Department of Neurology-CHR Citadelle, University of Liège, Belgium; 6 Catholic University of S. Cuore, Rome, Italy

## Background

Recent experimental evidence points out a possible involvement of outer and inner retinal layers in hypersensitivity of migraine patients to light stimuli. To investigate the short-wavelength-sensitive (S) and the medium/long-wavelength-sensitive (ML) cone photoreceptors of the visual pathways in migraine without aura (MO) patients between attacks and in healthy volunteers (HV) by using yellow-blue (Y-B) or red-blue (R-B) visual flicker stimuli.

## Methods

Square-wave focal electroretinograms (FERGs) were recorded in 22 MO patients and 20 HV. For each randomly presented flicker stimulation protocol (Y-B or R-B), 600 sweeps (4 Hz repetition rate) were recorded and partitioned in 6 blocks of 100. Fourier analysis allowed extracting from the FERG data the fundamental (1F) and the second harmonic (2F) components (amplitude and phase) that are related respectively to outer and inner retinal activity. Usual headache severity and photophobia during migraine were scored on a 0 to 10 visual analogue scale.

## Results

When compared to HV, MO patients had an advanced 1F phase but normal amplitude in all blocks of Y-B FERG. In MO patients, the self-rated intensity of ictal photophobia was positively correlated with attack frequency (r = 0.571, p = 0.01), headache severity (r = 0.508, p = 0.03), 1F Y-B phase (all blocks r = 0.487, p = 0.04), 1F R-B phase (r = 0.521, p = 0.03), 2F Y-B amplitude (all r = 0.610, p < 0.01), habituation slope (r = 0.686, p < 0.01), and 2F R-B phase (r = 0.526, p = 0.03).

## Conclusions

These results suggest that an abnormal signal transduction from the outer to the inner retinal layers could contribute to the mechanisms by which light causes pain or discomfort during the migraine headache.

Written informed consent to publication was obtained from the patient(s).

